# Redox-Modulations of Photophysical and Single-molecule Magnet Properties in Ytterbium Complexes Involving Extended-TTF Triads

**DOI:** 10.3390/molecules25030492

**Published:** 2020-01-23

**Authors:** Bertrand Lefeuvre, Jessica Flores Gonzalez, Frédéric Gendron, Vincent Dorcet, François Riobé, Vladimir Cherkasov, Olivier Maury, Boris Le Guennic, Olivier Cador, Viacheslav Kuropatov, Fabrice Pointillart

**Affiliations:** 1 Univ Rennes, CNRS, ISCR (Institut des Sciences Chimiques de Rennes)—UMR 6226, 35000 Rennes, France; bertrand.lefeuvre@univ-rennes1.fr (B.L.); jessica.flores-gonzales@univ-rennes1.fr (J.F.G.); frederic.gendron@univ-rennes1.fr (F.G.); vincent.dorcet@univ-rennes1.fr (V.D.); boris.leguennic@univ-rennes1.fr (B.L.G.); olivier.cador@univ-rennes1.fr (O.C.); 2 Univ Lyon, Ens de Lyon, CNRS UMR 5182, Université Claude Bernard Lyon 1, Laboratoire de Chimie, F69342 Lyon, France; francois.riobe@ens-lyon.fr (F.R.); olivier.maury@ens-lyon.fr (O.M.); 3 G. A. Razuvaev Institute of Organometallic Chemistry of Russian Academy of Sciences, GSP-445, Tropinina str. 49, 603950 Nizhny Novgorod, Russia; cherkasov@iomc.ras.ru

**Keywords:** ytterbium, extended-tetrathiafulvalene, electro-activity, single-molecule magnet, luminescence

## Abstract

The reaction between the 2,2’-benzene-1,4-diylbis(6-hydroxy-4,7-di-*tert*-butyl-1,3-benzodithiol-2-ylium-5-olate triad (**H_2_SQ**) and the metallo-precursor [Yb(hfac)_3_]⋅2H_2_O led to the formation of a dinuclear coordination complex of formula [Yb_2_(hfac)_6_(**H_2_SQ**)]⋅0.5CH_2_Cl_2_ (**H_2_SQ-Yb**). After chemical oxidation of **H_2_SQ** in 2,2’-cyclohexa-2,5-diene-1,4-diylidenebis(4,7-di-*tert*-butyl-1,3-benzodithiole-5,6-dione (**Q**), the latter triad reacted with the [Yb(hfac)_3_]⋅2H_2_O precursor to give the dinuclear complex of formula [Yb_2_(hfac)_6_(**Q**)] (**Q-Yb**). Both dinuclear compounds have been characterized by X-ray diffraction, DFT optimized structure and electronic absorption spectra. They behaved as field-induced Single-Molecule Magnets (SMMs) nevertheless the chemical oxidation of the semiquinone to quinone moieties accelerated by a factor of five the relaxation time of the magnetization of **Q-Yb** compared to the one for **H_2_SQ-Yb**. The **H_2_SQ** triad efficiently sensitized the Yb^III^ luminescence while the chemical oxidation of **H_2_SQ** into **Q** induced strong modification of the absorption properties and thus a quenching of the Yb^III^ luminescence for **Q-Yb**. In other words, both magnetic modulation and luminescence quenching are reached by the oxidation of the protonated semiquinone into quinone.

## 1. Introduction

Molecular magnetism is in intense expansion since the discovery of the first Single-Molecule Magnet (SMM) behavior for a Mn_12_ cluster [1] and a decade later the similar magnetic behavior for a mononuclear lanthanide complex [2]. The motivation for this kind of nanomagnets comes from the potential applications in high density data storage [3,4] especially since the development of organometallic chemistry allowing the discovery of “high blocking temperature” SMM [5,6,7,8]. The magnetic properties of the lanthanide ions strongly depend on the crystal field and slight variations of the electronic distribution of the lanthanides’ surrounding, making such ions excellent candidates for magnetic switches [9,10,11,12,13,14,15,16,17,18]. Among the possible magnetic property switches, those playing with oxidation-reduction reactions are the most promising because such a stimulus can lead to drastic modifications in the electronic properties of the molecular system. Oxidation-reduction reactions can be achieved thanks to redox-active metal centers [19,20,21] or/and organic ligands [22,23,24]. Lanthanides are also well-known for their luminescence ranging from the visible to the near infrared (NIR) region [25,26]. The sensitization of their emission is achieved by antenna effect involving singlet [27,28] and triplet [29,30,31] states of a (metallo)organic chromophore which can be energetically changed by oxido-reduction reaction leading to redox commutation of the NIR luminescence [32,33,34,35].

**Scheme 1:**
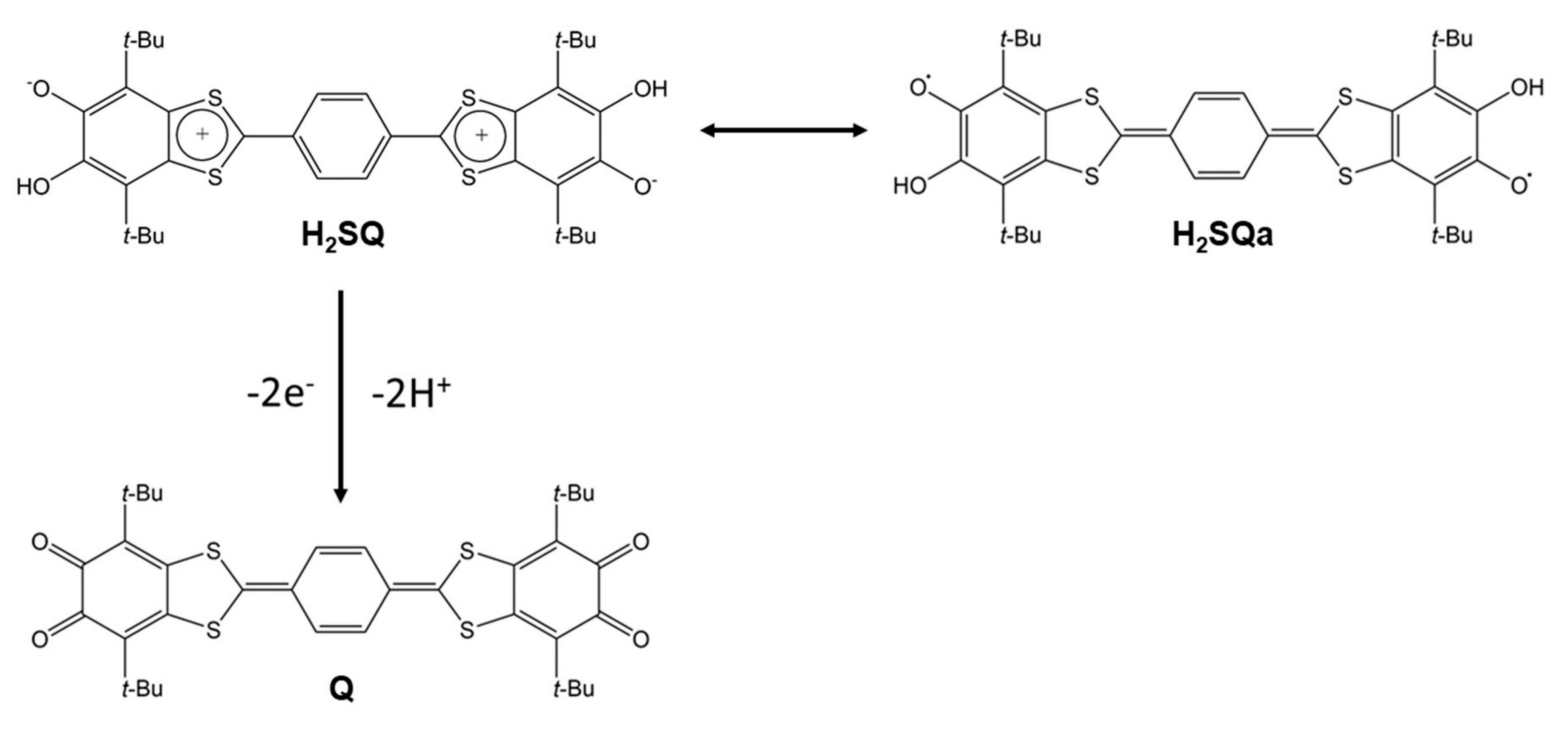
Molecular structures of the **H_2_SQ** and **Q** ligands and intramolecular electron transfer in **H_2_SQ**.

In this article, we proposed to exploit the ligand 2,2’-benzene-1,4-diylbis(6-hydroxy-4,7-di-*tert-*butyl-1,3-benzodithiol-2-ylium-5-olate [36] (**H_2_SQ**) (Scheme 1), which is suitable for lanthanide coordination, [37] and its oxidized form 2,2’-cyclohexa-2,5-diene-1,4-diylidenebis(4,7-di-*tert*-butyl-1,3-benzodithiole-5,6-dione (**Q**) (Scheme 1) to modulate both photophysical and magnetic properties of their corresponding complexes [Yb_2_(hfac)_6_(**H_2_SQ**)]⋅0.5CH_2_Cl_2_ (**H_2_SQ-Yb**) and [Yb_2_(hfac)_6_(**Q**)] (**Q-Yb**) (hfac^−^ = 1,1,1,5,5,5-hexafluororacetylacetonate).

## 2. Results and Discussion

### 2.1. X-Ray Structures

The coordination reaction of the 2,2’-benzene-1,4-diylbis(6-hydroxy-4,7-di-*tert*-butyl-1,3-benzodithiol-2-ylium-5-olate triad (**H_2_SQ**) (Scheme 1) and tris(1,1,1,5,5,5-hexafluoro-acetylacetonate)bis(aqueous)Yb^III^ (Yb(hfac)_3_⋅2H_2_O) in CH_2_Cl_2_ led to the formation of the dinuclear complex of formula [Yb_2_(hfac)_6_(**H_2_SQ**)]⋅0.5CH_2_Cl_2_ (**H_2_SQ-Yb**). The oxidized form of **H_2_SQ** was obtained using an excess of MnO_2_, the resulting **Q** ligand (Scheme 1) was immediately reacted with Yb(hfac)_3_⋅2H_2_O leading to the dinuclear complex [Yb_2_(hfac)_6_(**Q**)] (**Q-Yb**).

#### 2.1.1. [Yb_2_(hfac)_6_(H_2_SQ)]⋅0.5CH_2_Cl_2_ (**H_2_SQ-Yb**)

**Figure 1:**
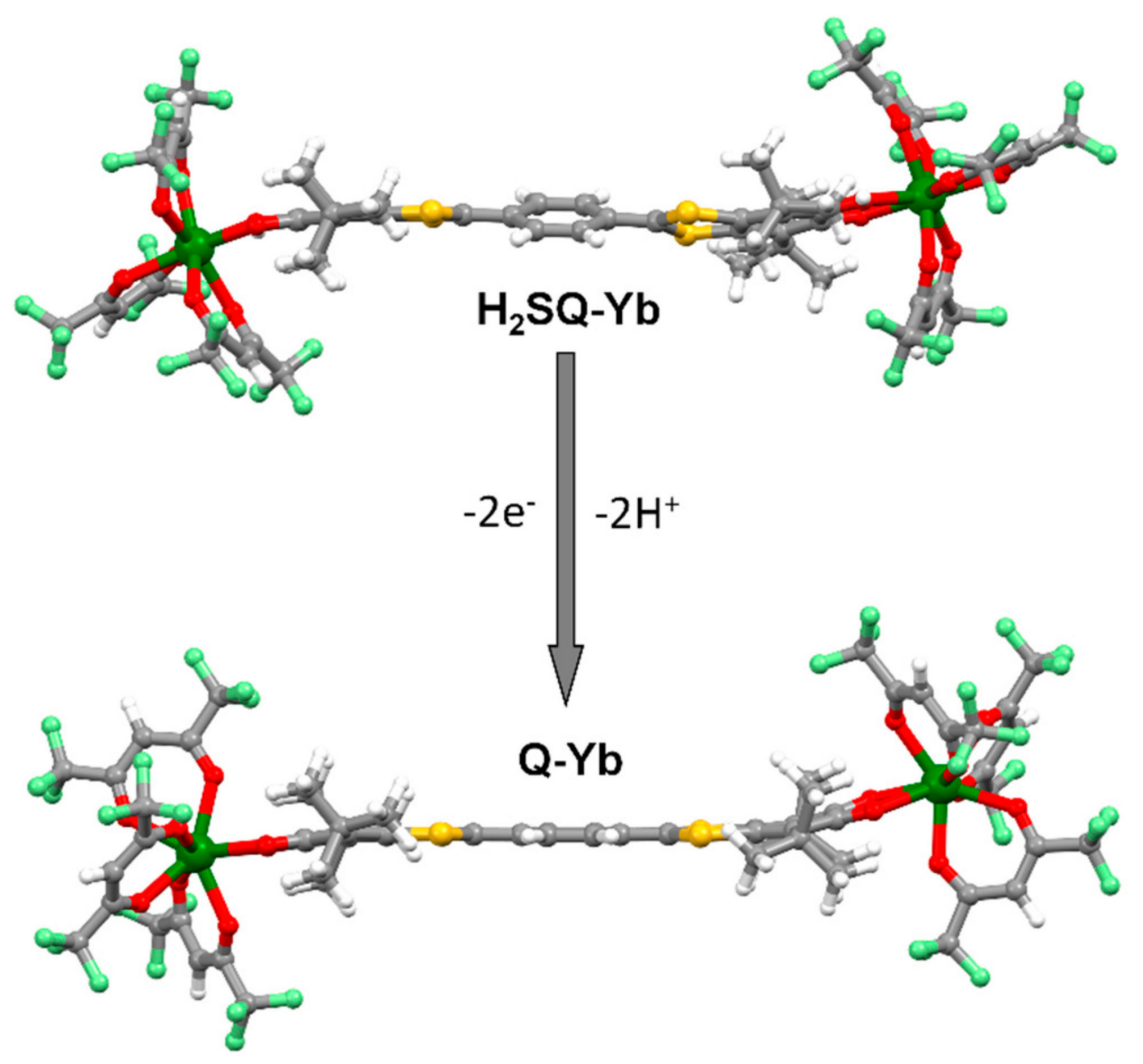
Experimental molecular structure of **H_2_SQ-Yb** and DFT optimized structure of its oxidized form **Q-Yb**. Molecules of crystallization are omitted for clarity.

**H_2_SQ-Yb** crystallizes in the monoclinic space group P2_1_/c (No. 14) (Figure 1 and Figure S1, Table S1). The asymmetric unit is composed by one molecule of [Yb_2_(hfac)_6_(**H_2_SQ**)] and one half dichloromethane molecule of crystallization. The Yb(hfac)_3_ units are linked to the two terminal monoprotonated semiquinone coordination sites. A bischelating coordination mode is observed through both C-O^−^ and C-OH groups (Figure 1). The coordination strength with the C-O^−^ is stronger than the one with C-OH as attested by the Yb-OH distances (2.408(5) Å) which are much longer than the Yb-O^−^ distances (2.178(4) Å). The limit case in which the coordination reaction takes place only through the C-O^−^ group has been recently observed [37]. The analysis of the C-OH (1.374(7) Å) and C-O^−^ (1.295(7) Å) bond lengths agrees with the protonated form of the semiquinone moieties. In order to confirm the oxidation state of the triad, the bond lengths of the ligand are carefully analyzed since an intramolecular electron transfer can take place from **H_2_SQ** to **H_2_SQa** (Scheme 1). The 1,3-dithiole rings in **H_2_SQ-Yb** is essentially aromatic since the S1-C15 (1.692(6) Å), S2-C15 (1.660(6) Å), S3-C22 (1.673(7) Å) and S4-C22 (1.671(6) Å) bond lengths are comparable with those for TTF dications (1.670-1.690 Å) while they are longer in neutral TTF (1.730–1.760 Å) [38,39,40]. The dicationic character of the extended TTF induced a single character of the C15-C16 and C21-C22 bonds as observed with the distances of 1.445(8) Å and 1.458(8) Å. The bond lengths distribution in the terminal six-membered rings in bridging ligand in **H_2_SQ-Yb** is also different from typical o-quinone distribution. The shortest distances in these rings were observed for bonds connecting *t*-Bu-substituted and carbonyl carbon atoms.

Finally, the protonated semiquinone form of the triad with charge-separated structure is also confirmed by the non-planarity of the ligand since the central *p*-phenylene ring and the two 6-hydroxy-4,7-di-*tert-*butyl-1,3-benzodithiol moieties formed a torsion angle of 26.2(2)° in agreement with the one found in the X-ray structure of the free **H_2_SQ** ligand [36]. Thus each Yb^III^ ion is surrounded by eight oxygen atoms coming from the three hfac^-^ anions and the bischelating **H_2_SQ** ligand. The coordination sphere around the metal center can be described as distorted triangular dodecahedron (D_2d_ symmetry) (SHAPE analysis, Table S2) [41].

**Figure 2:**
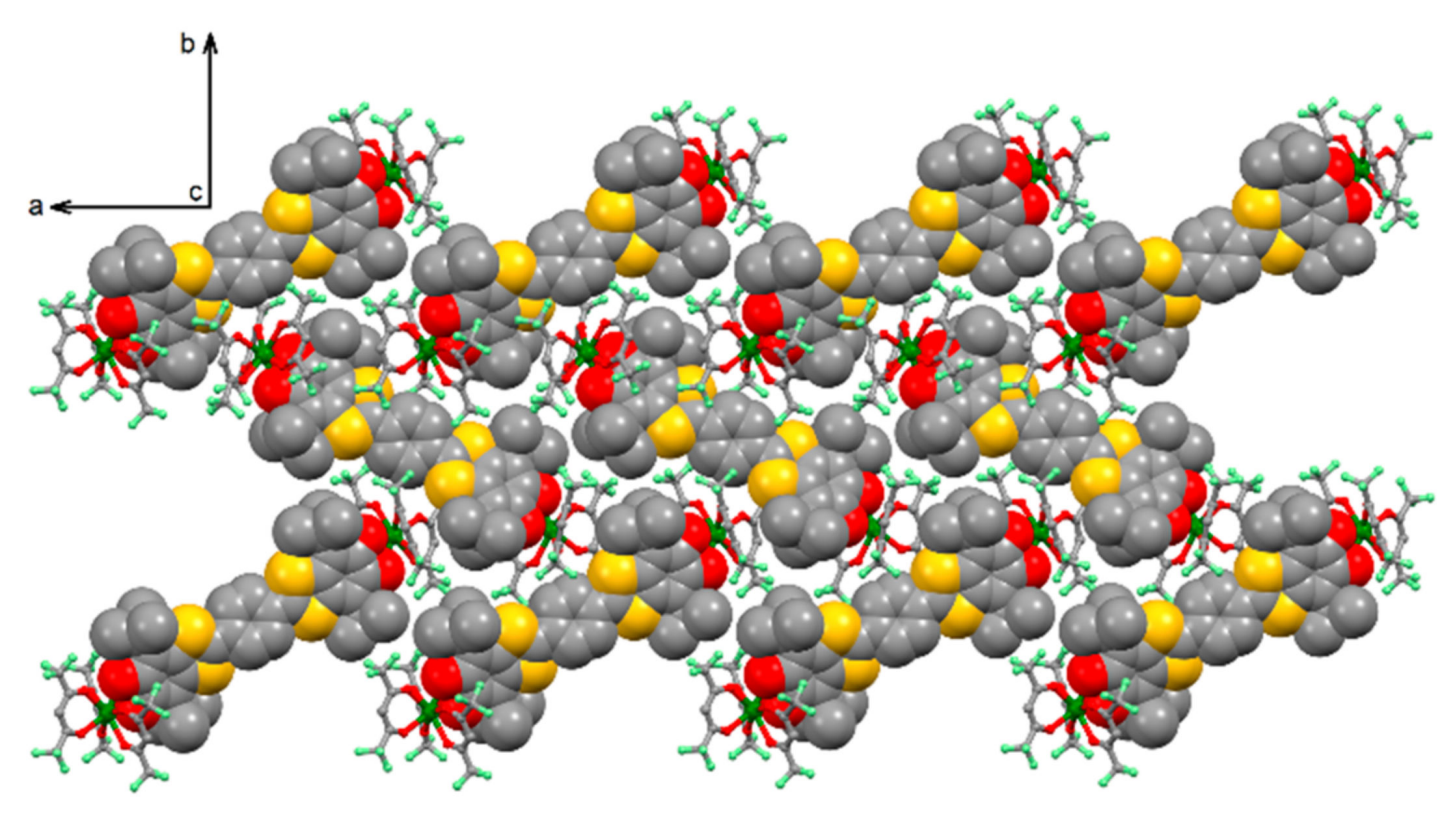
Crystal packing of **H_2_SQ-Yb** in the (110) plane. “Spacefill” and “ball and sticks” representations are used for **H_2_SQ** ligands and Yb(hfac)_3_ units, respectively.

The crystal packing reveals a good isolation of the two inorganic and organic sub-networks since nor π-π interaction either S⋅⋅⋅S contact are identified (Figure 2) which is quite unusual for this kind of π-extended sulfured systems. The integrity of the crystal is assumed by H⋅⋅⋅F interactions. The Yb-Yb intramolecular distance is 21.477 Å while the shortest Yb-Yb intermolecular distance is 10.000 Å.

#### 2.1.2. [Yb_2_(hfac)_6_(**Q**)] (**Q-Yb**)

Despite all the attempts to obtain suitable single crystals of **Q-Yb** for diffraction studies, we did not succeed. Consequently based on the X-ray structure of a previous system associated Yb(hfac)_3_ unit and quinone-based tetrathiafulvalene ligand [42], the structure of **H_2_SQ-Lu** and **Q-Lu** (Figure 1) have been optimized with the help of DFT (see Experimental Section for computational details and Table S4). The X-ray structure of **H_2_SQ-Yb** is well reproduced by the DFT calculations despite the replacement of the Yb(III) ions by diamagnetic Lu(III) ions. Only a minor lengthening of the metal-ligand bond lengths is calculated in **H_2_SQ-Lu** when compared to **H_2_SQ-Yb** (see Table S4). In **Q-**Lu, the two quinone moieties act as bischelating coordination site. The average six Lu-O_hfac_ distances (2.319 Å) are shorter than the average two Lu-O_quinone_ ones (2.360 Å) leading to distorted square antiprism (D_4d_) coordination environment around the Lu^III^ ions (SHAPE analysis, Table S2). The quinone form is confirmed by i) the C=O bond length of 1.267 Å shorter than the C-OH and C-O^−^ observed for the protonated semiquinone form and in agreement with the quinone form previously observed for quinone-based TTF and ii) with the almost planar shape of the **Q** ligand (torsion angle of 3.7°) because of the increasing of the aromaticity character of the ligand after oxidation. The coordination of the Yb(hfac)_3_ units plays a crucial role in the stabilization of the **Q** ligand since it was previously established that such an oxidized form of the free ligand cannot be isolated in solid-state due to its instability [36]. The Lu-Lu intramolecular distance is 21.664 Å.

### 2.2. Magnetic Properties

#### 2.2.1. Static Magnetic Measurements

**Figure 3:**
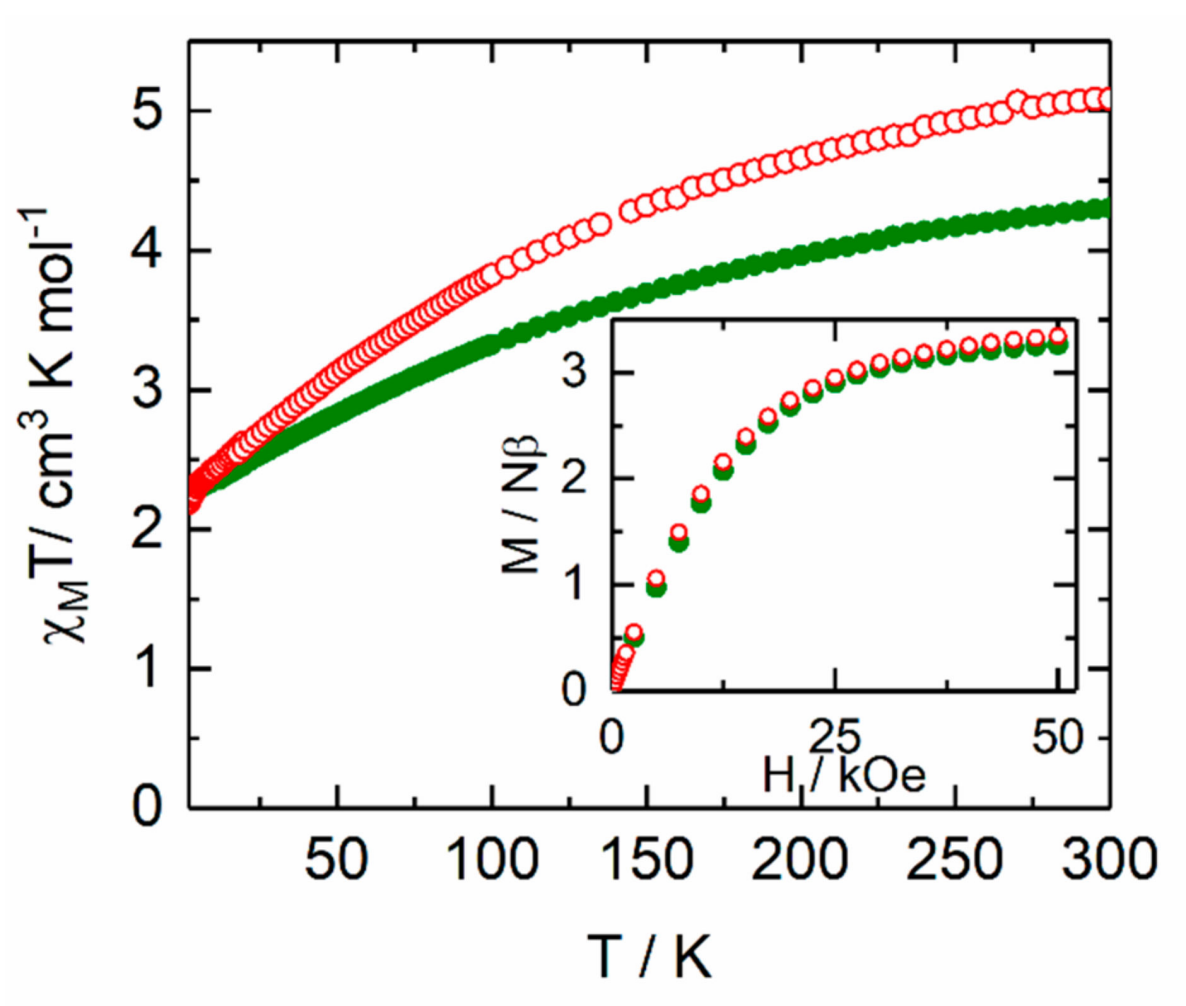
Temperature dependences of χ_M_T for **H_2_SQ-Yb** (green) and **Q-Yb** (red) in applied magnetic field of 10 kOe between 20 and 300 K while a magnetic field of only 2 kOe was used between 2 and 20 K. In inset, the field variations of the magnetization at 2 K for **H_2_SQ-Yb** (green) and **Q-Yb** (red) between 0 and 50 kOe.

The temperature dependence of *χ_M_T* for the samples **H_2_SQ-Yb** and **Q-Yb** are given for two Yb^III^ centers and represented in Figure 3. The room temperature values are 4.30 cm^3^·K·mol^−1^ and 5.08 cm^3^ K mol^−1^ for **H_2_SQ-Yb** and **Q-Yb**, respectively.

Such values are in the range of observed values for Yb^III^-based molecular systems [43] and in agreement with the expected value for two Yb^III^ ions (5.14 cm^3^ K mol^−1^, ^2^F_7/2_ ground state multiplet) [44]. Upon cooling, *χ_M_T* decreases monotonically down to 2.24 cm^3^·K·mol^−1^ and 2.19 cm^3^ K mol^−1^ at 2 K for **H_2_SQ-Yb** and **Q-Yb**, respectively. Taking into account the intra- and intermolecular distance between the metal centers, the decrease of the *χ_M_T* products is attributed to the thermal depopulation of the M_J_ states. **H_2_SQ-Yb** and **Q-Yb** exhibited similar magnetization with experimental values of 3.26 Nβ and 3.34 Nβ at 50 kOe, respectively [44].

#### 2.2.2. Dynamic Magnetic Measurements

**Figure 4:**
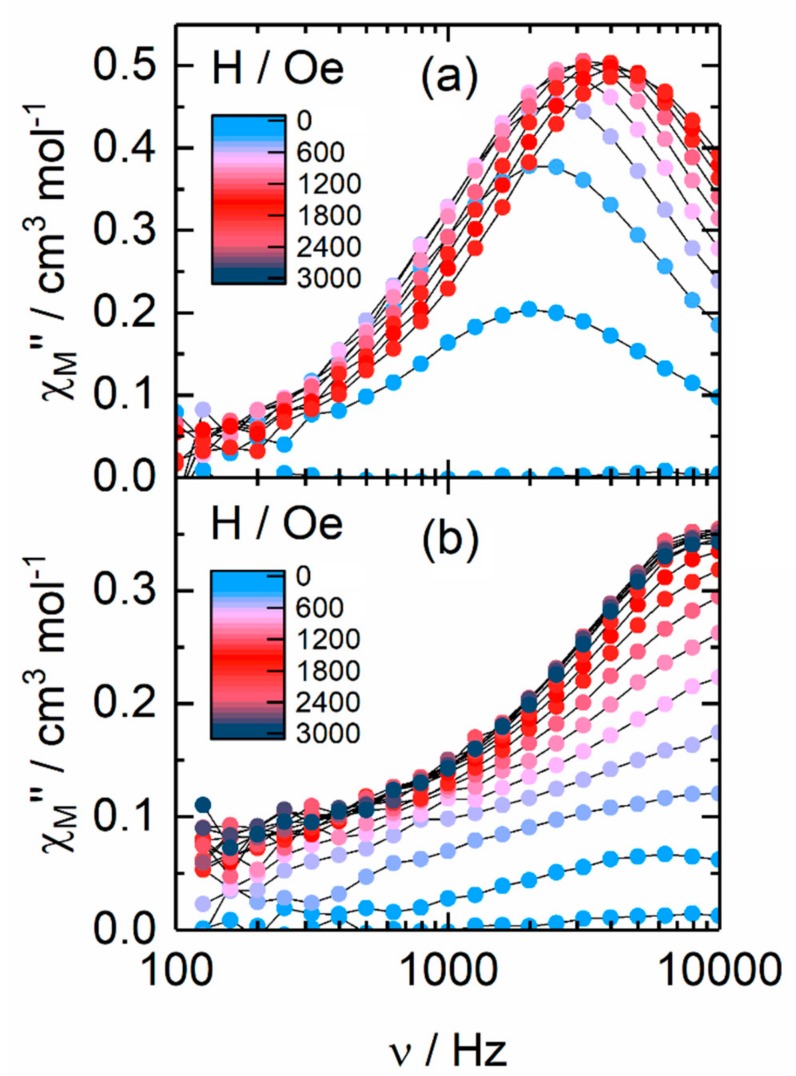
Frequency dependence of χ_M_” between 0 and 3000 Oe for **H_2_SQ-Yb** (**a**) and **Q-Yb** (**b**) at 2K.

The in-phase and out-of-phase components of the ac susceptibility (χ_M_’’) for both compounds **H_2_SQ-Yb** and **Q-Yb** were measured using immobilized selected and crunched single crystals. For both compounds, any out-of-phase signal was not detected in zero magnetic field in the 1–10000 Hz frequency range. Such absence of out-of-phase component of the ac susceptibility in 0 Oe is common for the Yb^III^-based coordination complexes because of the fast magnetic relaxation due to efficient Quantum Tunneling of the Magnetization (QTM) which can be cancelled by applying an external magnetic field [45]. Thus the field dependence of the magnetic susceptibility is studied in detail (Figure 4 and Figure S2). For both compounds, the application of a small magnetic field led to the appearance of out-of-phase component of the magnetic susceptibility and the optimal value of the applied magnetic field was chosen thank to the field dependence of the relaxation time (Figure S3). The scan field revealed an unusual behavior since applying a small magnetic field led to the slowest magnetic relaxation of a fraction of the sample (about 50 %) while increasing the value of the applied magnetic field accelerates the magnetic relaxation but increases the relaxing fraction. In fact applying a magnetic field tends to cancel the QTM (appearance of the slow magnetic relaxation fraction) but favor the appearance of field dependent under energy barrier mechanism (direct process). Thus a good compromised magnetic field value could be 800 Oe for **H_2_SQ-Yb** while for such field value the maximum was observed at the highest accessible frequency range for **Q-Yb** at 2 K (Figure 4 and Figure S2).

At this point one can notice that the oxidation of the **H_2_SQ** ligand is accompanied by a fastening of the magnetic relaxation by a factor 5 at 2 K.

**Figure 5:**
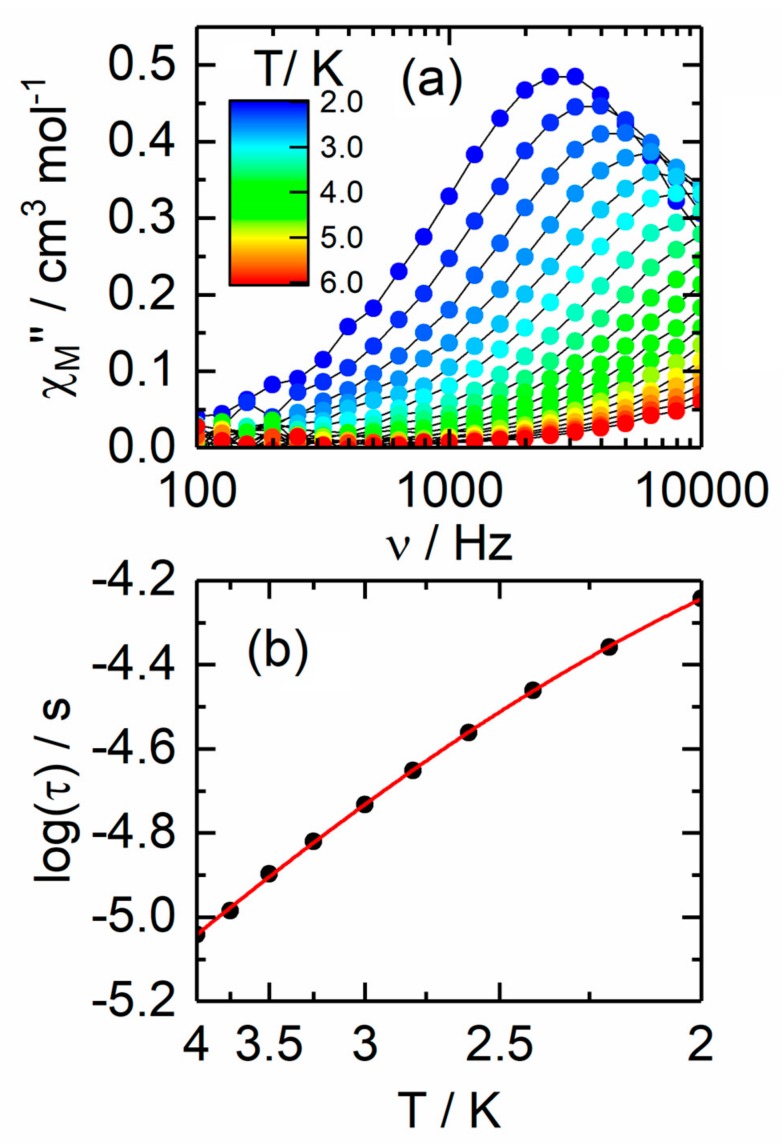
(**a**) Frequency dependence of χ_M_” between 2 and 6 K for **H_2_SQ-Yb** at 800 Oe, (**b**) Temperature variation of the relaxation time for **H_2_SQ-Yb** in the temperature range of 2–4 K with the best fitted curve with the Arrhenius law (red line).

Under an applied field of H = 800 Oe, **H_2_SQ-Yb** highlighted a frequency dependence of the out-of-phase signal of the magnetization (Figure 5a and Figure S4) which can be analyzed in the framework of the extended Debye model [46,47,48]. The temperature dependence of the relaxation time is extracted (see Supplementary Information for details) and depicted in Figure 5b (Table S3) and the normalized Argand (Figure S5) concluded that at such magnetic field more than 90 % of the sample was slowly relaxing. The relaxation time follows two thermally dependent Orbach and direct processes of relaxation:

τ^−1^ = τ_0_^−1^exp(Δ/T) + BTH^m^ (1)

The best fit was obtained using the Equation (1) with τ_0_ = 9.25(50) × 10^−8^ s and Δ = 10.0(2) K, and B = 1.23(4) × 10^−8^ s^−1^ K^−1^ Oe^−4^ and m = 4 (fixed) where Δ is the energy barrier, B and m are constant parameters for the direct relaxation process (Figure 5b). The involvement of direct process is strongly supported by the field dependence of the relaxation time (Figures S2 and S3) and the selected 800 Oe applied magnetic field. All the other attempts to include other magnetic relaxation processes led to a predominant Raman mechanism which exclude both remaining QTM and Direct process (Figures S6–S8). Nevertheless, the field dependence of the magnetic susceptibility (Figures S2–S3) imposed the presence of the Direct process.

#### 2.2.3. Photophysical Properties

**Figure 6:**
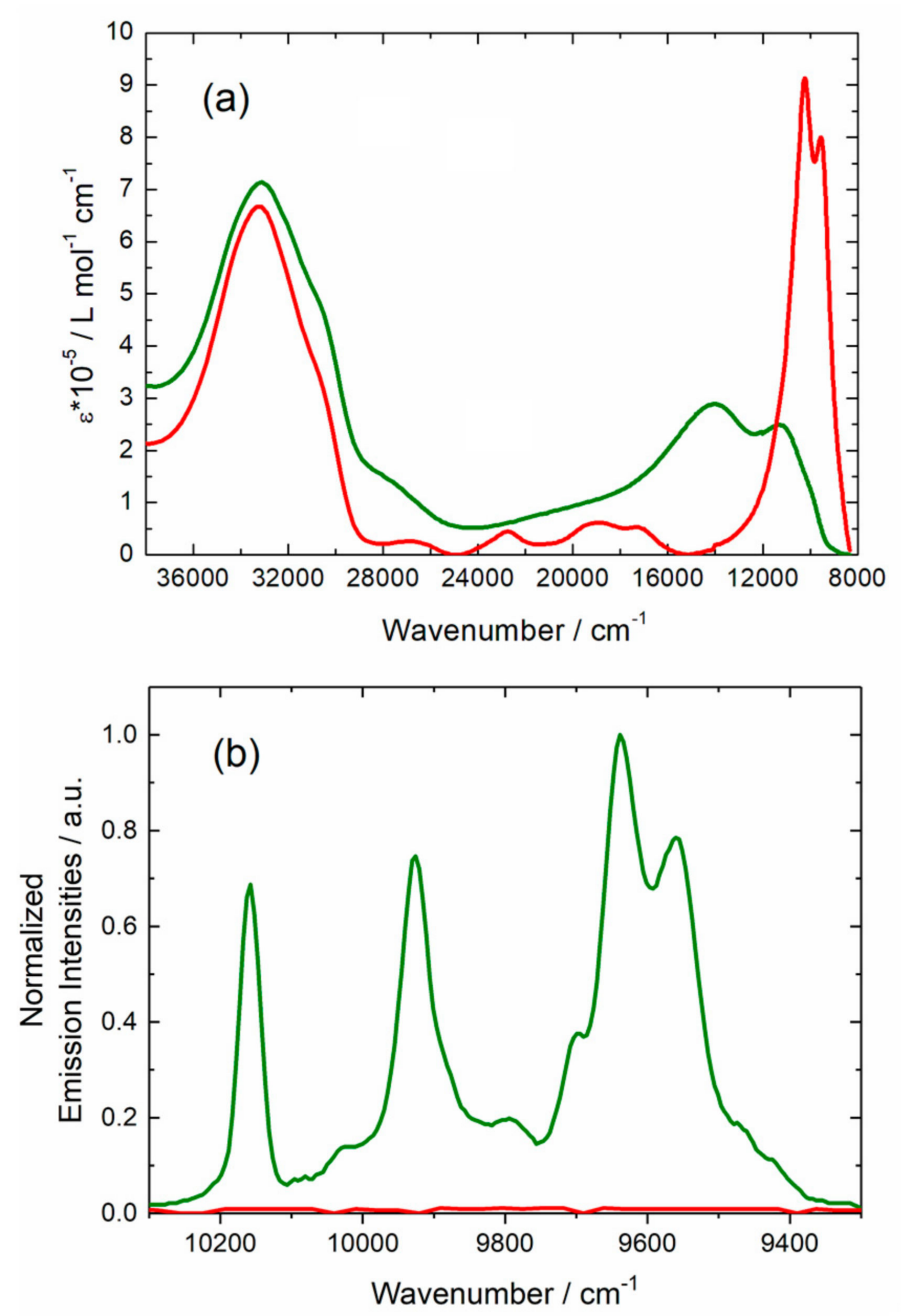
(**a**) Experimental UV-visible absorption spectra at room temperature of **H_2_SQ-Yb** (green line) and **Q-Yb** (red line) in CH_2_Cl_2_ solution (C = 4 × 10^−5^ mol.L^−1^). (**b**) Emission spectra at 77 K of **H_2_SQ-Yb** (green line) and **Q-Yb** (red line) for an irradiation energy of 16670 cm^−1^ in solid-state.

The UV-visible absorption properties for **H_2_SQ-Yb** and **Q-Yb** have been studied in a CH_2_Cl_2_ solution (Figure 6a). The spectra are dominated by the π-π* hfac^−^ transition at high energy (33000 cm^−1^) and Intra-Ligand Charge Transfers (ILCTs) at low energy. S → L and H → L (where S = Single Occupied Molecular Orbital, L = Lowest Unoccupied Molecular Orbital and H = Highest Occupied Molecular Orbital) ILCT have been previously identified for the corresponding free **H_2_SQ** and **Q** triads with the S and H mainly localized on the extended TTF while L is situated on the semiquinone and quinone acceptors [36,49,50]. Thus for **H_2_SQ-Yb** and based on DFT calculations for the free **H_2_SQ**triad [36], the strong absorption band centered at 12500 cm^−1^ is attributed to the H → L ILCT and the shoulder centered at 20.000 cm^−1^ is attributed to S → L ILCT. For **Q-Yb**, the most intense absorption band at 9800 cm^−1^ is attributed to the H → L ILCT. The oxidation of the **H_2_SQ-Yb** into **Q-Yb** induced a redshift of the H → L ILCT from 12500 cm^−1^ to 9800 cm^−1^ (average energy value of the maxima). It was previously demonstrated that the low-energy ILCT could be an efficient donating source for sensitization of NIR lanthanide emitters [27,51,52]. Irradiation at 16670 cm^–1^ (or lower energy) induced the expected ^2^F_5/2_ → ^2^F_7/2_ Yb^III^ emission (Figure 6b) for **H_2_SQ-Yb** whereas after the two-electron oxidation the same irradiation highlighted the total quenching of the NIR emission for **Q-Yb**. The NIR luminescence of **H_2_SQ-Yb** is composed of four components as expected from the splitting of the ^2^F_7/2_ ground state by the ligand field effect [53,54,55] localized at 10157 cm^−1^, 9926 cm^–1^, 9639 cm^−1^ and 9560 cm^−1^ (Figure 6b). Since the emission of the lanthanide ions can be interpreted as a photography of the ground state energy splitting, the experimental energy barrier determined from the energy gap between the ground and the first excited states is 231 cm^−1^ (332 K) which is much more higher than the value determined from the magnetic properties (10 K). Such observation may signify that the Orbach contribution is negligible and the magnetic relaxation occurs mainly through the direct process.

**Scheme 2:**
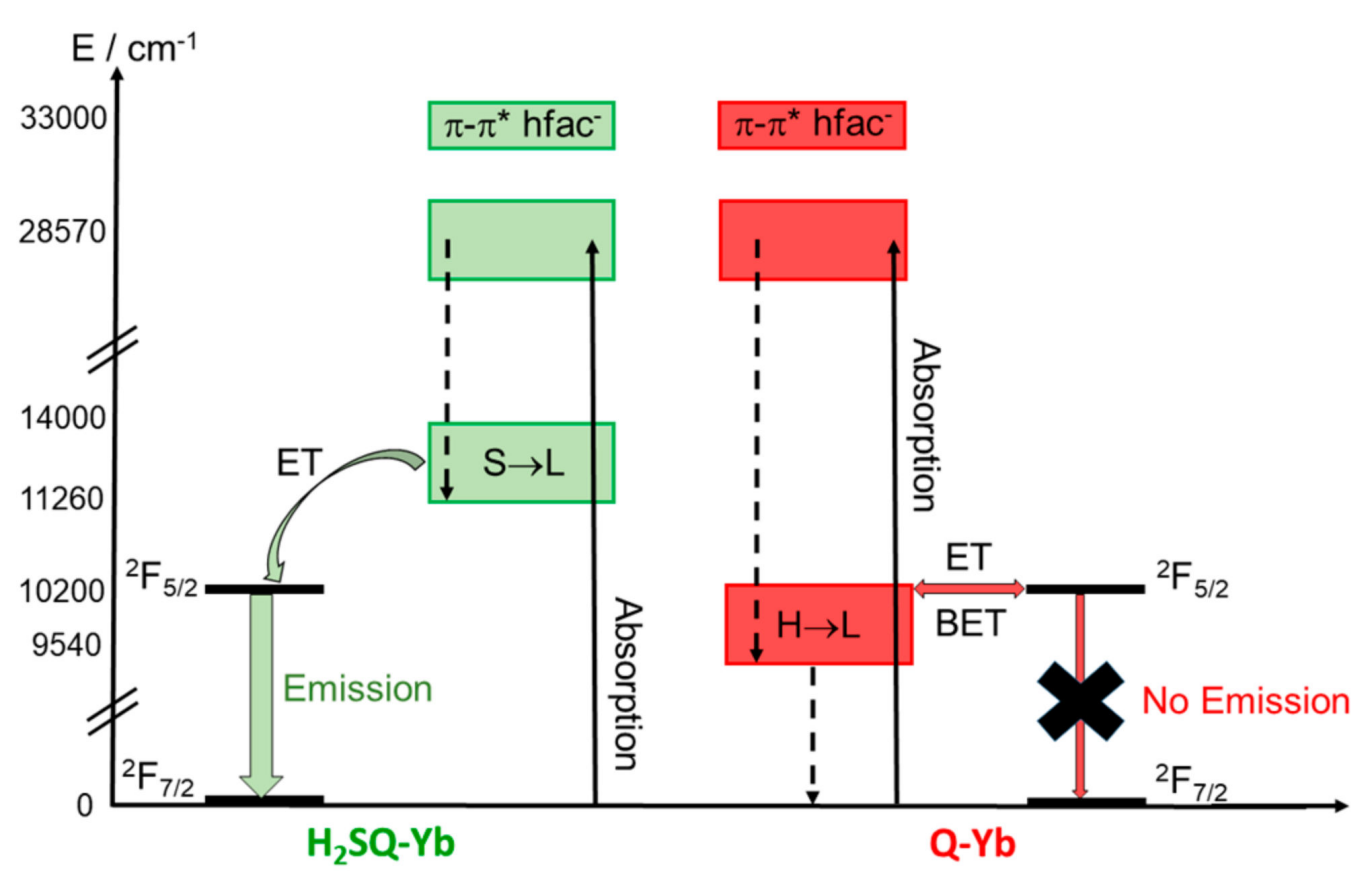
Proposed sensitization process in **H_2_SQ-Yb** (left part) and **Q-Yb** (right part). Dashed lines represent the non-radiative deactivations, ET = Energy Transfer, BET = Back Energy Transfer, H = HOMO, L = LUMO and S = SOMO.

The efficiency of the NIR emission sensitization strongly depends on the energy position of the ILCT. The donating ILCT (S→L in Scheme 2) in **H_2_SQ-Yb** is localized 2300 cm^−1^ above the emitting ^2^F_5/2_ level which is well suited for an efficient sensitization while both donating and emitting levels are localized at the same energy in **Q-Yb** leading to efficient back energy transfer (BET) and thus a quenching of the NIR emission.

## 3. Materials and Methods

### 3.1. Synthesis. General Procedures and Materials

The precursor Dy(hfac)_3_⋅2H_2_O (hfac^−^ = 1,1,1,5,5,5-hexafluoroacetonate anion) and the 2,2’-benzene-1,4-diylbis(6-hydroxy-4,7-di-*tert*-butyl-1,3-benzodithiol-2-ylium-5-olate ligand (**H_2_SQ**) were synthesized following previously reported methods [36,56]. All other reagents were commercially available and used without further purification.

### 3.2. Synthesis of complexes [Yb_2_(hfac)_6_(H_2_SQ)]⋅0.5CH_2_Cl_2_ (H_2_SQ-Yb) and [Yb_2_(hfac)_6_(Q)] (Q-Yb)

*[Yb_2_(hfac)_6_(**H_2_SQ**)]⋅0.5CH_2_Cl_2_ (**H_2_SQ-Yb**)*. 66.4 mg of Yb(hfac)_3_⋅2H_2_O (0.08 mmol) were dissolved in 10 mL of CH_2_Cl_2_ and then added to a purple solution of 10 mL of CH_2_Cl_2_ containing 26.4 mg of **H_2_SQ** (0.04 mmol). The purple solution of **H_2_SQ** immediately turned blue with the addition of the Yb^III^ salt. After 15 minutes of stirring, 20 mL of *n*-hexane were layered at room temperature in the dark. Slow diffusion leads to dark blue single crystals of **H_2_SQ-Yb** which are suitable for X-ray studies. Yield (determined from isolated single crystals) 40.4 mg (44 %). Anal. Calcd (%) for C_66.5_H_49_Yb_2_F_36_O_16_S_4_Cl: C 37.73, H 2.13; found: C 38.09, H 2.22.

*[Yb_2_(hfac)_6_(**Q**)] (**Q-Yb**)*. 13.2 mg of **H_2_SQ** (0.02 mmol) were dissolved in 20 mL of CH_2_Cl_2_ and then stirred in presence of 1.5 g of MnO_2_. The starting purple solution turned green (oxidation of **H_2_SQ** into **Q**) and after 45 min of stirring it was filtered directly in a CH_2_Cl_2_ solution (5 mL) of Yb(hfac)_3_⋅2H_2_O (33.2 mg, 0.04 mmol). The green solution turned to a dark pink color. Addition of *n*-pentane into the resulting dark pink solution led to formation of a dark pink crystalline powder. Yield 32.0 mg (71 %). Anal. Calcd (%) for C_66_H_46_Yb_2_F_36_O_16_S_4_: C 35.18, H 2.04; found: C 35.66, H 2.19.

### 3.3. Crystallography

Single crystal of **H_2_SQ-Yb** was mounted on a APEXIII D8 VENTURE AXS diffractometer for data collection (Bruker, Billerica, MA, USA), MoK_α_ radiation source, λ = 0.71073 Å), from the Centre de Diffractométrie (CDIFX), Université de Rennes 1, France (Table S1). Structure was solved with a direct method using the SHELXT program [57] and refined with a full matrix least-squares method on F^2^ using the SHELXL-14/7 program [58]. Complete crystal structure results as a CIF file including bond lengths, angles, and atomic coordinates are deposited as Supporting Information. CCDC number is 1970010 for compound **H_2_SQ-Yb**.

### 3.4. Physical Measurements

The elemental analyses of the compounds were performed at the Centre Régional de Mesures Physiques de l’Ouest, Rennes, France. Absorption spectra were recorded on a V-650 spectrophotometer (JASCO, Easton, MD, USA) in dilute solutions, using spectrophotometric grade solvents. Emission spectra were measured using Fluorolog-3 fluorimeter (Horiba-Jobin–Yvon, Kyoto, Japan). The steady-state luminescence was excited by unpolarized light from a 450 W xenon continuous wave (CW) lamp and detected at an angle of 90° by a nitrogen-cooled InGAs detector (Horiba-Jobin–Yvon, Kyoto, Japan). Quartz tubes containing solid samples of each compound were place in a quartz dewar containing liquid nitrogen The dc magnetic susceptibility measurements were performed on solid polycrystalline samples with a MPMS-XL SQUID magnetometer (Quantum Design Inc., San Diego, CA, USA) between 20 and 300 K in applied magnetic field of 2 kOe between 2 and 20 K and 10 kOe above. The microcrystallites are immobilized in a pellet made with Teflon tape. These measurements were all corrected for the diamagnetic contribution as calculated with Pascal’s constants. The ac magnetic susceptibility measurements were performed on a Quantum Design PPMS (Quantum Design Inc., San Diego, CA, USA) system equipped with ac/dc probe.

### 3.5. Computational Details

The structure optimization of **Q-Yb** was carried out at the Kohn-Sham density functional theory (DFT) level with the Amsterdam Density Functional (ADF) [59,60,61] software package. In this calculation, the Yb^3+^ center was replaced by the closed-shell Lu^3+^ ion, and the scalar all-electron zeroth-order regular approximation (ZORA) [62] was employed for the treatment of the scalar relativistic effects. The PBE functional (Perdew-Burke-Ernzerhof) [63] from the generalized gradient approximation, was used along with the triple-ζ polarized Slater0type orbital (STO) all-electron basis set with one set of polarization function for all atoms (TZP) [64].

## 4. Conclusions

In this article, the 2,2’-benzene-1,4-diylbis(6-hydroxy-4,7-di-*tert*-butyl-1,3-benzodithiol-2-ylium-5-olate triad (**H_2_SQ**) allowed the formation of a dinuclear complex of formula [Yb_2_(hfac)_6_(**H_2_SQ**)]⋅0.5CH_2_Cl_2_ (**H_2_SQ-Yb**) in which the two Yb^III^ ions are coordinated to the bischelating protonated semiquinone units. After the chemical oxidation of the **H_2_SQ** triad, the resulting 2,2’-cyclohexa-2,5-diene-1,4-diylidenebis(4,7-di-*tert*-butyl-1,3-benzodithiole-5,6-dione **Q** triad allowed the formation of a new dinuclear complex [Yb_2_(hfac)_6_(**Q**)] (**Q-Yb**). The two-electron oxidation of the triad induced changes in the electronic absorption spectra with a red shift of the ILCT bands. For **H_2_SQ-Yb**, irradiation of the ILCT induced an efficient sensitization of the Yb^III^ NIR emission while the lower-energy ILCT for **Q-Yb** allowed back energy transfer and so a quenching of the NIR emission. Both compounds behave as field-induced SMM with a fastening of the magnetic relaxation after oxidation. In conclusion the oxidation of the **H_2_SQ** triad into the **Q** one induced a dual magnetic modulation and luminescence quenching.

## Supplementary Materials

The following are available online at https://www.mdpi.com/1420-3049/25/3/492/s1, Figure S1. ORTEP view of the asymmetric unit in [Yb_2_(hfac)_6_(H_2_SQ)]⋅0.5CH_2_Cl_2_ (**H_2_SQ-Yb**). Thermal ellipsoids are drawn at 30% probability. Hydrogen atoms and CH_2_Cl_2_ molecule of crystallization are omitted for clarity. Figure S2. Scan field of the frequency dependence of the in phase (χ_M_’) component of the ac magnetic susceptibility for **H_2_SQ-Yb** (a) and **Q-Yb** (b). Figure S3. Field dependence of the magnetic relaxation time of **H_2_SQ-Yb**. Figure S4. Frequency dependence of the in-phase component of the magnetic susceptibility for **H_2_SQ-Yb** measured under a DC applied magnetic field of 800 Oe in the 2-6 K temperature range. Figure S5. Normalized Cole-Cole plots for **H_2_SQ-Yb** at several temperatures between 2 and 3 K. Table S1: X-ray crystallographic data for **H_2_SQ-Yb**. Table S2: SHAPE analysis of the coordination polyhedra around the lanthanide in **H_2_SQ-Yb** and **Q-Yb**.; Table S3: Best fitted parameters (χ_T_, χ_S_, τ and α) with the extended Debye model for compound **H_2_SQ-Yb** at 800 Oe in the temperature range 2-4 K.
