# Supplementary material for: Redox-Modulations of Photophysical and Single-molecule Magnet Properties in Ytterbium Complexes Involving Extended-TTF Triads
Source: Molecules. 2020 Jan 23;25(3):492. doi: 10.3390/molecules25030492 (PMC7038133; doi:10.3390/molecules25030492)
Supplement: Supplementary file 1 [file molecules-25-00492-s001.pdf]

molecules

MDPI

Supplementary Materials

Redox-Modulations of Photo-physical and Single-Molecule Magnet Properties in Ytterbium Complexes Involving Extended-TTF Triads

Bertrand Lefeuvre [1], Jessica Flores Gonzalez [1], Frédéric Gendron [1], Vincent Dorcet [1], François Riobé [2], Vladimir Cherkasov [3], Olivier Maury [2], Boris Le Guennic [1], Olivier Cador [1], Viacheslav Kuropatov [3,*] and Fabrice Pointillart [1,*]

[1] Univ Rennes, CNRS, ISCR (Institut des Sciences Chimiques de Rennes) - UMR 6226, 35000 Rennes, France; bertrand.lefeuvre@univ-rennes1.fr (B.L.); jessica.flores-gonzales@univ-rennes1.fr (J.F.G.); frederic.gendron@univ-rennes1.fr (F.G.); vincent.dorcet@univ-rennes1.fr (V.D.); boris.leguennic@univ-rennes1.fr (B.L.G.); olivier.cador@univ-rennes1.fr (O.C.)

[2] Univ Lyon, Ens de Lyon, CNRS UMR 5182, Université Claude Bernard Lyon 1, Laboratoire de Chimie, F69342, Lyon, France; francois.riobe@ens-lyon.fr (F.R.); olivier.maury@ens-lyon.fr (O.M.)

[3] G. A. Razuvaev Institute of Organometallic Chemistry of Russian Academy of Sciences, 603950, GSP-445, Tropinina str., 49, Nizhny Novgorod, Russia; cherkasov@iomc.ras.ru

* Correspondence: viach@iomc.ras.ru (V.K.); Tel: +79-(0)51902995 and fabrice.pointillart@univ-rennes1.fr (F.P.); Tel.: +33-(0)223236752

Academic Editors: Dawid Pinkowicz and Robert Podgajny
Received: 20 December 2019; Accepted: 22 January 2020; Published: date

Abstract: The reaction between the 2,2′-benzene-1,4-diylbis(6-hydroxy-4,7-di-*tert*-butyl-1,3-benzodithiol-2-ylium-5-olate triad (**H_2SQ**) and the metallo-precursor [Yb(hfac)$_3$]·2H_2O led to the formation of a dinuclear coordination complex of formula [Yb$_2$(hfac)$_6$(**H_2SQ**)]·0.5CH_2Cl_2 (**H_2SQ-Yb**). After chemical oxidation of **H_2SQ** in 2,2′-cyclohexa-2,5-diene-1,4-diylidenebis(4,7-di-*tert*-butyl-1,3-benzodithiole-5,6-dione (**Q**), the latter triad reacted with the [Yb(hfac)$_3$]·2H_2O precursor to give the dinuclear complex of formula [Yb$_2$(hfac)$_6$(**Q**)] (**Q-Yb**). Both dinuclear compounds have been characterized by X-ray diffraction, DFT optimized structure and electronic absorption spectra. They behaved as field-induced Single-Molecule Magnets (SMMs) nevertheless the chemical oxidation of the semiquinone to quinone moieties accelerated by a factor of five the relaxation time of the magnetization of **Q-Yb** compared to the one for **H_2SQ-Yb**. The **H_2SQ** triad efficiently sensitized the Yb^{III} luminescence while the chemical oxidation of **H_2SQ** into **Q** induced strong modification of the absorption properties and thus a quenching of the Yb^{III} luminescence for **Q-Yb**. In other words, both magnetic modulation and luminescence quenching are reached by the oxidation of the protonated semiquinone into quinone.

Keywords: ytterbium; extended-tetrathiafulvalene; electro-activity; single-molecule magnet; luminescence

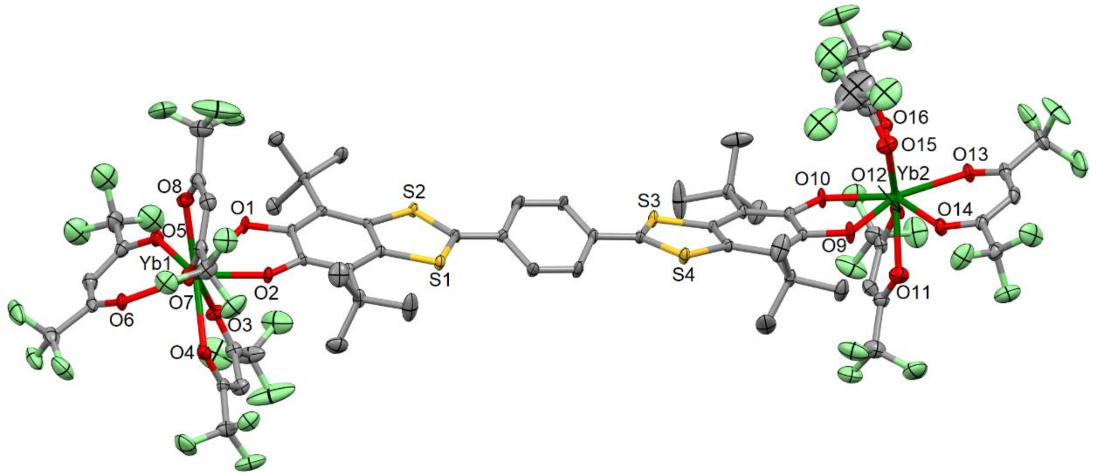

Figure S1. ORTEP view of the asymmetric unit in $[Yb_2(hfac)_6(\mathbf{H_2SQ})]\cdot 0.5CH_2Cl_2$ (**H_2SQ-Yb**). Thermal ellipsoids are drawn at 30% probability. Hydrogen atoms and CH_2Cl_2 molecule of crystallization are omitted for clarity.

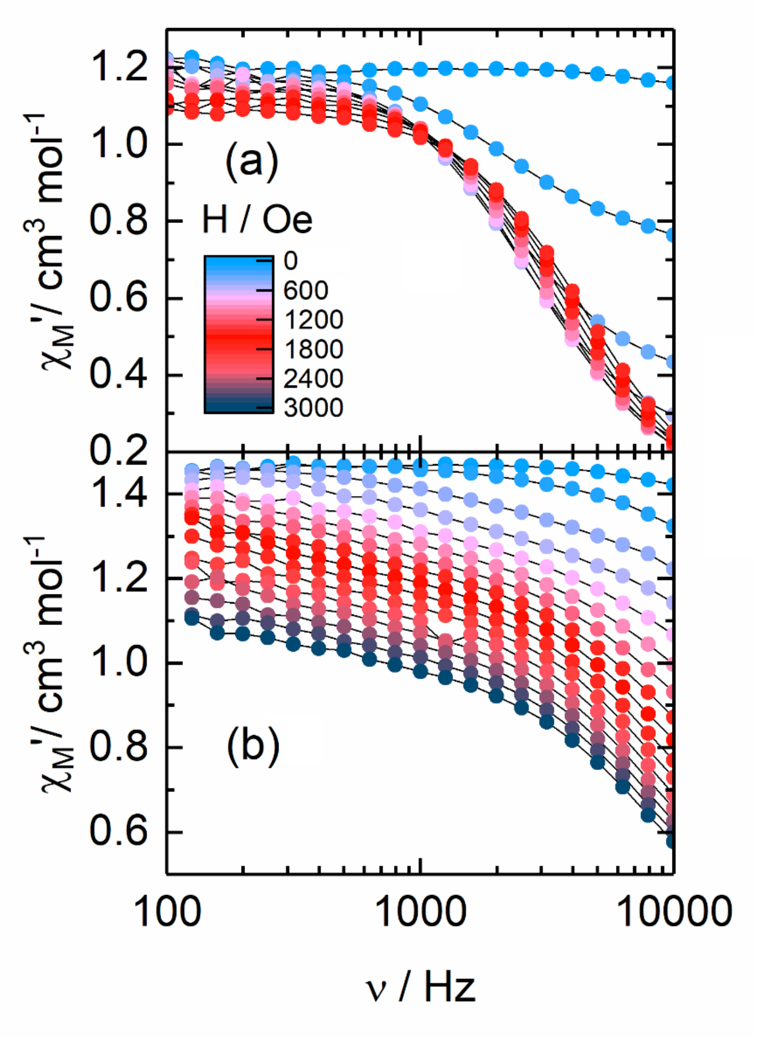

Figure S2. Scan field of the frequency dependence of the in phase (χ_M') component of the ac magnetic susceptibility for **H_2SQ-Yb** (a) and **Q-Yb** (b).

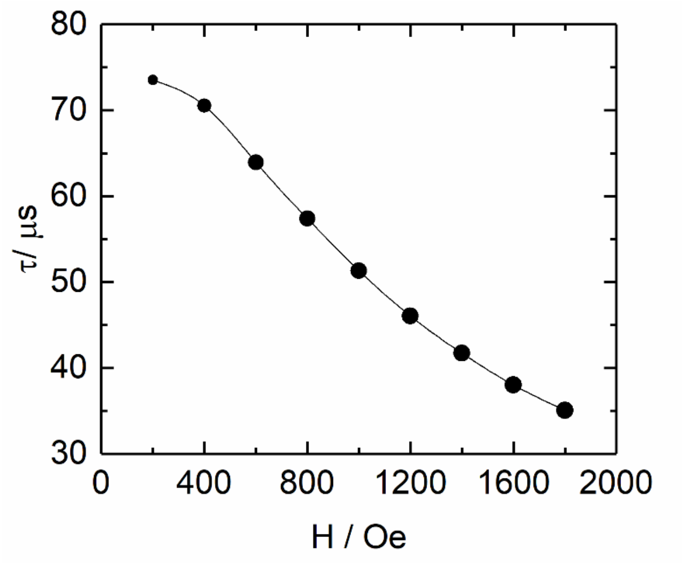

Figure S3. Field dependence of the magnetic relaxation time of **H_2SQ-Yb.** The size of the dots is proportional to the fraction of the sample (from 50% at 200 Oe to 100 % at 1600 Oe).

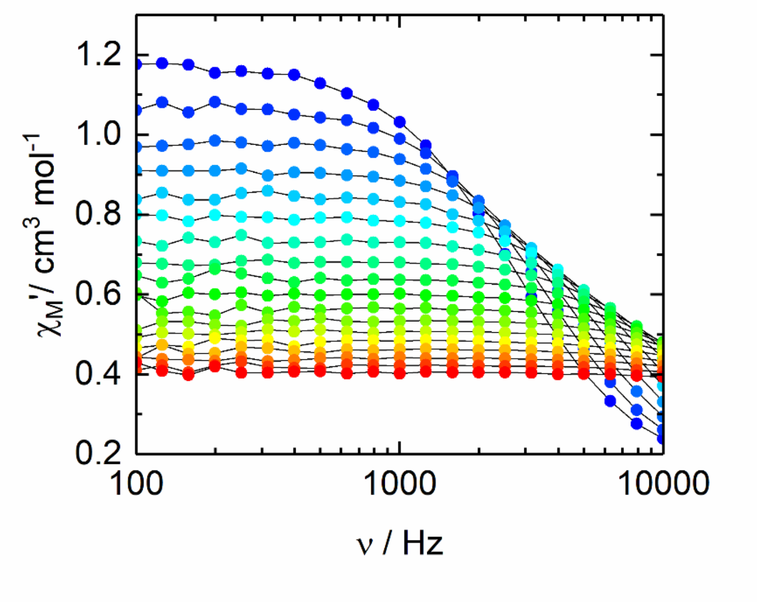

Figure S4. Frequency dependence of the in-phase component of the magnetic susceptibility for **H_2SQ-Yb** measured under a DC applied magnetic field of 800 Oe in the 2-6 K temperature range.

Extended Debye model.

$$\chi_M' = \chi_S + (\chi_T - \chi_S)\frac{1+(\omega\tau)^{1-\alpha}\sin\left(\alpha\frac{\pi}{2}\right)}{1+2(\omega\tau)^{1-\alpha}\sin\left(\alpha\frac{\pi}{2}\right)+(\omega\tau)^{2-2\alpha}}$$

$$\chi_M'' = (\chi_T - \chi_S)\frac{(\omega\tau)^{1-\alpha}\cos\left(\alpha\frac{\pi}{2}\right)}{1+2(\omega\tau)^{1-\alpha}\sin\left(\alpha\frac{\pi}{2}\right)+(\omega\tau)^{2-2\alpha}}$$

With χ_T the isothermal susceptibility, χ_S the adiabatic susceptibility, τ the relaxation time and α an empiric parameter which describe the distribution of the relaxation time. For SMM with only one relaxing object α is close to zero. The extended Debye model was applied to fit simultaneously the experimental variations of χ_M' and χ_M'' with the frequency f of the oscillating field ($\omega = 2\pi f$). Typically, only the temperatures for which a maximum on the χ'' vs. f curves, have been considered. The best fitted parameters τ, α, χ_T, χ_S are listed in Table S3 with the coefficient of determination R^2.

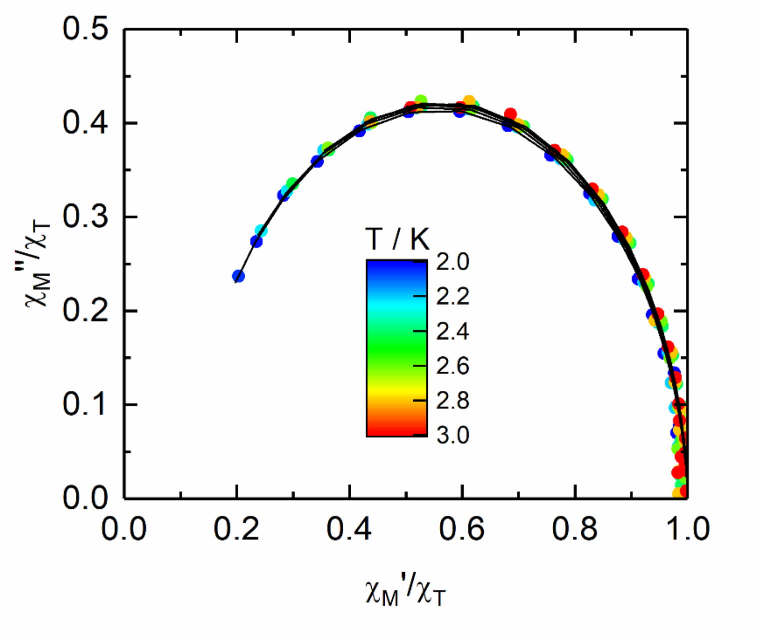

Figure S5. Normalized Cole-Cole plots for **H_2SQ-Yb** at several temperatures between 2 and 3 K.

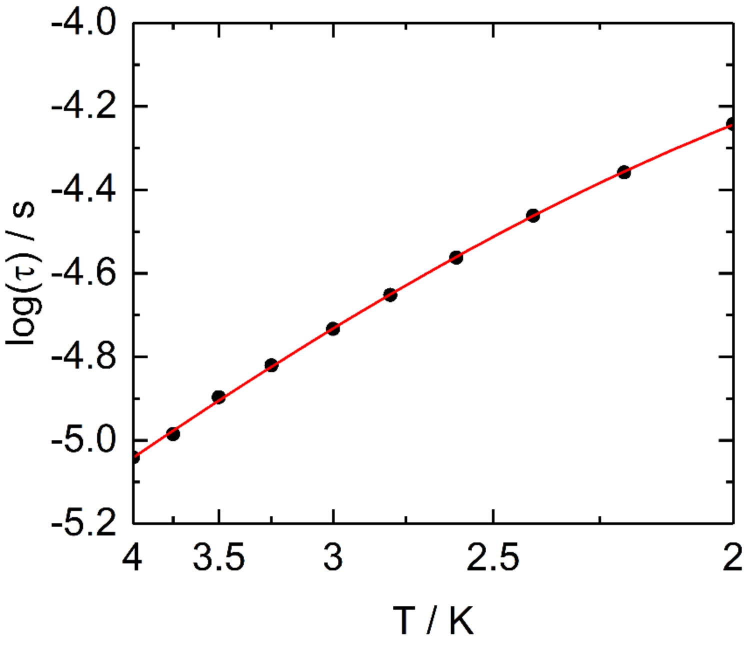

Figure S6. Temperature variation of the relaxation time for **H_2SQ-Yb** in the temperature range of 2-4 K with the best fitted curve with the Arrhenius law (red line). The best fit was obtained for the combination of Orbach + direct + QTM processes of relaxation ($\tau^{-1} = \tau_0^{-1}\exp(\Delta/T) + BTH^m + \tau_{TI}^{-1}$) with the following parameters with $\tau_0 = 8.91(53)\times10^{-7}$ s, $\Delta = 10.1(3)$ K, and $B = 1.28(8)\times10^{-8}$ s^{-1} K^{-1} Oe^{-4} and m = 4 (fixed) and $\tau_{TI} = 1.177\times10^{11}$ s.

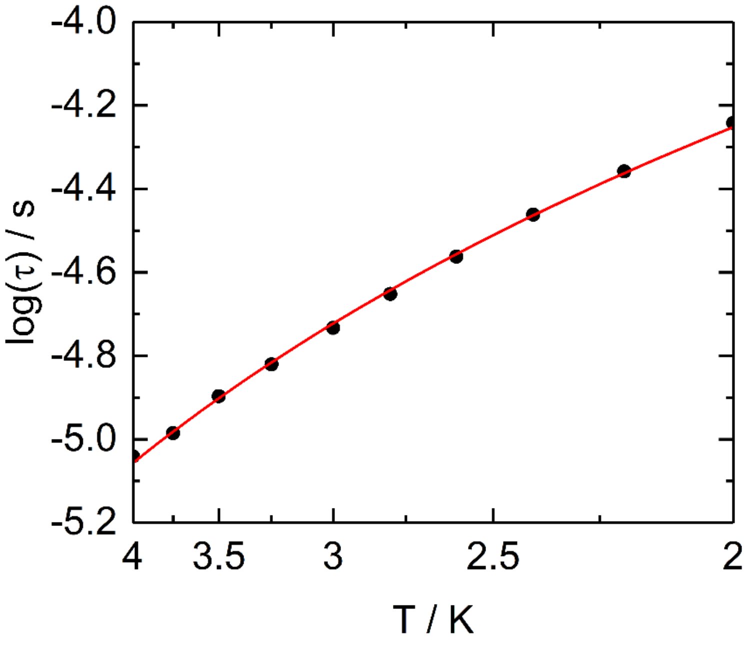

Figure S7. Temperature variation of the relaxation time for **H_2SQ-Yb** in the temperature range of 2-4 K with the best fitted curve with the Arrhenius law (red line). The best fit was obtained for the combination of Raman + direct processes of relaxation ($\tau^{-1} = CT^n + BTH^m$) with the following parameters with B = 0 s^{-1} K^{-1} Oe^{-4} and m = 4 (fixed) and C = 2800(1328) s^{-1} $K^{2.67}$ with n = 2.67(27).

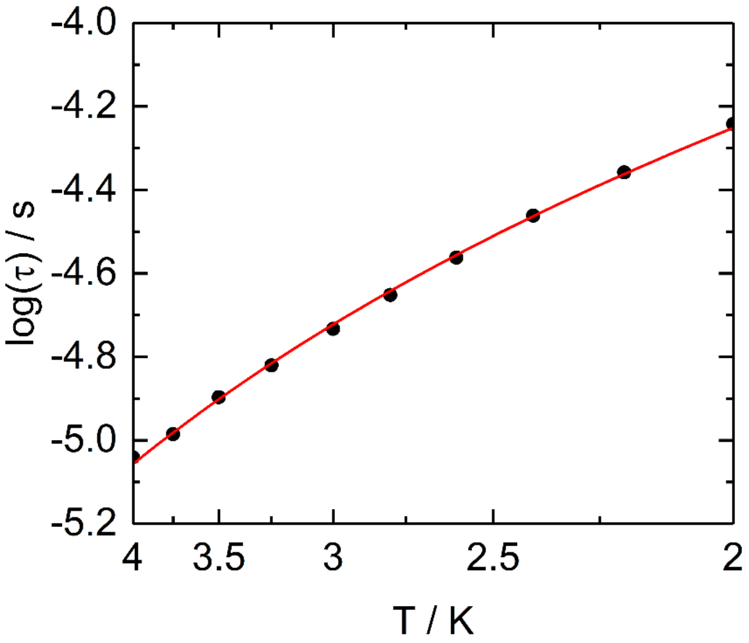

Figure S8. Temperature variation of the relaxation time for **H_2SQ-Yb** in the temperature range of 2-4 K with the best fitted curve with the Arrhenius law (red line). The best fit was obtained for the combination of Raman + direct + QTM ($\tau^{-1} = CT^n + BTH^m + \tau_{TI}^{-1}$) processes of relaxation with the following parameters with B = 0 s^{-1} K^{-1} Oe^{-4} and m = 4 (fixed) and C = 2797 s^{-1} $K^{2.67}$ with n = 2.67 and τ_{TI} = $1.256{\times}10^{12}$ s.

Table S1. X-ray crystallographic data for **H_2SQ-Yb**.

**Compounds**	**[$Yb_2(hfac)_6(H_2SQ)$]·0.5CH_2Cl_2 (H_2SQ-Yb)**
Formula	$C_{66.5}H_{49}Yb_2F_{36}O_{16}S_4Cl$
M / g.mol^{-1}	2297.8
Crystal system	Monoclinic
Space group	$P2_1/c$ (N°14)
Cell parameters	a = 20.4105(18) Å<br>b = 22.8006(19) Å<br>c = 18.4142(16) Å<br>β = 94.556(4)°
Volume / Å^3	8542.4(13)
Z	4
T / K	150 (2)
2θ range /°	$5.92 \leq 2\theta \leq 54.97$
ρcalc / g.cm^{-3}	1.787
μ / mm^{-1}	2.443
Number of reflections	88911
Independent reflections	19036
Rint	0.0942
$Fo^2 > 2\sigma(Fo)^2$	12361
Number of variables	1127
R1, ωR2	0.0536, 0.1108

Table S2. SHAPE analysis of the coordination polyhedra around the lanthanide in **H_2SQ-Yb** and **H_2SQ-Lu and Q-Lu**.[1].

**Compounds**	**Metal**	**$CShM_{SAPR-8}$ (square antiprism D_{4d})**	**$CShM_{BTPR-8}$ (biaugmented trigonal prism C_{2v})**	**$CShM_{TDD-8}$ (triangular dodecahedron D_{2d})**
**H_2SQ-Yb**	Yb1	3.176	2.188	0.534
	Yb2	2.404	1.700	0.562
**H_2SQ-Lu**	Lu1	1.751	1.997	0.868
	Lu2	2.794	2.442	0.555
**Q-Lu**	Lu1	0.541	1.783	1.842
	Lu2	0.452	1.724	2.160

Table S3. Best fitted parameters (χ_T, χ_S, τ and α) with the extended Debye model for compound **H_2SQ-Yb** at 800 Oe in the temperature range 2-3 K.

T / K	χ_T / cm³ mol⁻¹	χ_S / cm³ mol⁻¹	α	τ / s	R^2
2	1.17703	0.13528	0.0418	5.72624×10^{-5}	0.99985
2.2	1.07343	0.12506	0.03514	4.38424×10^{-5}	0.99967
2.4	0.98259	0.12501	0.02111	3.45253×10^{-5}	0.99978
2.6	0.91353	0.10954	0.02857	2.73988×10^{-5}	0.99985
2.8	0.84966	0.10401	0.02566	2.2299×10^{-5}	0.99968
3.0	0.79618	0.09982	0.02367	1.84951×10^{-5}	0.99966
3.25	0.73596	0.10146	0.01587	1.51233E-5	0.99954
3.5	0.68147	0.10557	5.96747E-14	1.26781E-5	0.99956
3.75	0.64204	0.09154	0.01788	1.03494E-5	0.99938
4	0.59965	0.10048	4.99889E-16	9.09481E-6	0.99948

Table S4. Principal bond lengths (in angstrom) for the X-ray structure of **H_2SQ-Yb** and for the DFT optimized structures **H_2SQ-Lu** and **Q-Lu**.

	RX H_2SQ-Yb	DFT H_2SQ-Lu	DFT Q-Lu
M-$O_{quinone}$	2.178 - 2.411	2.226 - 2.493	2.334 - 2.385
< M-$O_{quinone}$ >	2.292	2.345	2.360
M-O_{hfac}	2.280 - 2.343	2.305 - 2.374	2.292 - 2.343
< M-O_{hfac} >	2.310	2.332	2.319
C=O	1.289 / 1.301	1.308 / 1.310	1.267
COH	1.372 / 1.375	1.375 / 1.388	
M-M	21.477	21.391	21.664

Reference

1. M. Llunell, D. Casanova, J. Cirera, J. M. Bofill, P. Alemany, S. Alvarez, S. SHAPE (version 2.1), Barcelona, 2013.

© 2020 by the authors. Submitted for possible open access publication under the terms and conditions of the Creative Commons Attribution (CC BY) license (http://creativecommons.org/licenses/by/4.0/).
